# Characterization of the Keratinolytic Activity of Three *Streptomyces* Strains and Impact of Their Co-Cultivation on This Activity

**DOI:** 10.3390/microorganisms11051109

**Published:** 2023-04-24

**Authors:** Diego Martín-González, Sergio Bordel, Fernando Santos-Beneit

**Affiliations:** 1 Institute of Sustainable Processes, Dr. Mergelina, s/n, 47011 Valladolid, Spain; 2 Department of Chemical Engineering and Environmental Technology, School of Industrial Engineering, University of Valladolid, Dr. Mergelina, s/n, 47011 Valladolid, Spain; 3 Department of Functional Biology, Medical School, University of Oviedo, Av. Julián Clavería, 6, 33006 Oviedo, Spain

**Keywords:** *Streptomyces*, undecylprodigiosin, *Staphylococcus aureus*, keratinase, antibiotic, feathers, biofertilizers, co-culture

## Abstract

In this study, we describe the characterization of three efficient chicken feather-degrading *Streptomyces* bacteria isolated from honeybee samples and assess the impact of their co-cultivation on this activity and antistaphylococcal activity. *Streptomyces griseoaurantiacus* AD2 was the strain showing the highest keratinolytic activity (4000 U × mL^−1^), followed by *Streptomyces albidoflavus* AN1 and *Streptomyces drozdowiczii* AD1, which both generated approximately 3000 U × mL^−1^. Moreover, a consortium constituted of these three strains was able to use chicken feathers as its sole nutrient source and growth in such conditions led to a significant increase in antibiotic production. *S. griseoaurantiacus* AD2 was the only strain that exhibited weak antimicrobial activity against *Staphylococcus aureus*. UPLC analyses revealed that a significant number of peaks detected in the extracts of co-cultures of the three strains were missing in the extracts of individual cultures. In addition, the production of specialized metabolites, such as undecylprodigiosin and manumycin A, was clearly enhanced in co-culture conditions, in agreement with the results of the antimicrobial bioassays against *S. aureus*. Our results revealed the benefits of co-cultivation of these bacterial species in terms of metabolic wealth and antibiotic production. Our work could thus contribute to the development of novel microbial-based strategies to valorize keratin waste.

## 1. Introduction

Promoting the circular economy is part of the United Nations Sustainable Development Goals, and is one of the pillars of the so-called “New Green Deal” of the European Union. Therefore, it is very important to characterize the potential of all living beings, including that of microorganisms to use and/or valorize natural resources that are considered waste. For example, globally, poultry farming produces thousands of tons of chicken feathers each year [1] and chicken feathers are considered waste since they cannot be used to feed animals, even if they have a potential nutritional value, or cannot be used in any other profitable application. Therefore, feathers are normally dumped in landfills or incinerated [2]. However, some microorganisms produce special enzymes, known as keratinases, that can degrade these keratin-rich wastes [3]. Previously, the isolation of strains from soil and honeybee products led to the characterization of three *Bacillus* strains with high keratinolytic activity [4,5]. These strains in addition to showing keratinolytic activity also exhibited other interesting properties, such as the capability to degrade plastics (*Bacillus altitudinis* B12 [4]) or to inhibit the growth of Gram-positive bacteria (*Bacillus licheniformis* CG1 and *Bacillus sonorensis* AB7, both isolated from honeybee products [5]). Among the three strains, the highest keratinolytic activity was achieved by *B. licheniformis* CG1 (3800 U × mL^−1^), followed by *B. altitudinis* B12 (1500 U × mL^−1^) and *B. sonorensis* AB7 (1450 U × mL^−1^).

*Streptomyces* species stand out as the most important producers of antibiotics among all genera of bacteria discovered to date [6]. In addition to antibiotics, most of these bacteria can produce other useful bioactive compounds, including anticancer, immunosuppressants, herbicides, insecticides, pigments and a wide variety of enzymes that can hydrolyze a huge number of different substrates [7]. We have also isolated *Streptomyces* species from honeybee samples and characterized their antimicrobial properties [8]. These species were directly collected either from raw honey (i.e., *S. drozdowiczii* AD1 and *S. griseoaurantiacus* AD2) or from bee pollen (i.e., *S. albidoflavus* AN1). Using Liquid Chromatography-Mass Spectrometry (LC-MS) and dereplication analyses (LC-DAD-HRMS), we were able to characterize two metabolites, the red undecylprodigiosin and the colorless manumycin A, that were responsible for the antibiotic activity of *S. griseoaurantiacus* AD2 against Gram-positive bacteria such as *Staphylococcus aureus*, *Enterococcus faecalis* and *Bacillus subtilis* [8]. Since these *Streptomyces* strains have been isolated from the same type of samples as the previously mentioned *Bacillus* isolates [5], which showed both keratinolytic properties and antimicrobial activity, in this study, we characterize the keratinolytic potential of these *Streptomyces* species that were previously characterized in terms of their antimicrobial potential but not on their capability to degrade keratin. We also assessed the impact of co-cultivation of these three *Streptomyces* strains on their ability to use chicken feathers as their sole nutrient source and to produce undecylprodigiosin and manumycin A using bioassays as well as LC-DAD-HRMS and LC-MS analyses. Our results suggest that microbial-based strategies to recycle and valorize keratin-rich wastes could be developed to yield useful antibiotic mixtures or novel biofertilizers.

## 2. Materials and Methods

### 2.1. Microbial Growth Conditions and Growth Determination

The *Streptomyces* strains used in this study were isolated from samples of honey and pollen that were collected from beehives located in South East England [8]. R5A medium [9] inoculated with 10^6^ spores per mL [10,11] was used for routine growth of the *Streptomyces* strains. Otherwise, *Streptomyces* cells were grown in a mineral salt medium with chicken feathers that we named CFMS (Chicken Feathers Mineral Salts). This medium contains glucose (1 g × L^−1^), peptone (0.5 g × L^−1^), KH_2_PO_4_ (0.7 g × L^−1^), K_2_HPO_4_ (1.4 g × L^−1^), MgSO_4_ (0.1 g × L^−1^), NaCl (0.5 g × L^−1^), ZnSO_4_·7H_2_0 (0.05 g × L^−1^), FeSO_4_·7H_2_0 (0.015 g × L^−1^) and chicken feathers (10 g × L^−1^) and had pH = 8; or on a medium that we named CFTW (Chicken Feathers Tap Water). This medium contains solely chicken feathers (10 g × L^−1^) and tap water (no carbon, nitrogen, or phosphate sources nor trace elements were added to this medium whose pH was also not adjusted). For the experiment on the effect of pH on keratinolytic activity, the pH of the CFMS medium was adjusted to 7 (neutral) or 6 (acid) instead of 8 (basic). In all cases, chicken feathers were provided by a poultry farm in Valladolid, Spain. The feathers were processed as follows: (i) washing with tap water and Triton-X to get rid of any debris, (ii) removal of Triton-X with distilled water, (iii) dried at 60 °C overnight, (iv) cut into small pieces for proper addition to the cultures. For the chicken feathers degradation experiment on CFTW medium, cultures were performed in 2.2 L bottles with 200 mL of medium and 1 mL of an R5A preculture with each of the *Streptomyces* species grown separately or together in a co-culture during an identical period of time (i.e., 48 h). The initial R5A pre-cultures of each individual *Streptomyces* strain were achieved by inoculation with 10^6^ spores per mL whereas that of the co-culture was inoculated with 10^2^ spores per mL of each strain yielding a total amount of 10^6^ spores per mL. To quantify bacterial growth and monitor the keratin degradation process during time-series experiments of several weeks, the cultures were performed in 2.2 L glass bottles hermetically closed with an isoprene rubber and aluminum crimp seal. Since the mycelial growth of the *Streptomyces* cells and the turbidity generated by the degradation of the feathers hampers the use of optical density to monitor growth, CO_2_ and O_2_ concentrations were quantified in the atmosphere of the hermetically closed bottles throughout the time-series experiments. Oxidation of carbohydrates through aerobic respiration consumed O_2_ and released CO_2_ at an equivalent ratio. O_2_ and CO_2_ concentrations were measured using a gas chromatograph (Agilent 8860, The Netherlands) equipped with a thermal conductivity detector (GC-TCD) following procedures described elsewhere [12].

### 2.2. Analysis of Metabolites by Ultra Performance Liquid Chromatography (UPLC)

Metabolites from the *Streptomyces* R5A cultures (inoculated with 10^6^ live spores) grown during 96 h (i.e., stationary phase) were extracted using the following procedure. Ethyl acetate was added to the samples (containing both supernatants and biomass) at a 1:1 ratio (20 mL:20 mL) and maintained in constant mixing for 2 h. The organic phase (≤20 mL) was separated from the aqueous phase by centrifugation and collected for further evaporation using a SpeedVac. The resulting extracts were re-suspended in 200 µL of methanol. The cell extracts were analyzed by reversed-phase chromatography using acetonitrile and water containing 0.1% trifluoroacetic acid (TFA) as solvents in an Acquity UPLC instrument fitted with a BEH C18 column (1.7 mm, 2.1 × 100 mm; Waters). Samples were eluted with 10% acetonitrile for 1 min, followed by a linear gradient from 10 to 100% acetonitrile over 7 min, at a flow rate of 0.5 mL min^−1^ and a column temperature of 35 °C. Detection and spectral characterization of the peaks obtained from UPLC was performed by photodiode array detection using Empower software v.3 (Waters) to extract bi-dimensional chromatograms at different wavelengths.

### 2.3. Analysis of Metabolites by Liquid Chromatography-Mass Spectrometry (LC-MS)

LC-MS analysis was performed using an Alliance chromatographic system coupled to a ZQ4000 mass spectrometer and a SunFire C18 column (3.5 mm, 2.1 × 150 mm; Waters). Solvents were the same as those used in the UPLC analyses and elution was performed with an initial isocratic hold with 10% acetonitrile for 4 min followed by a linear gradient from 10 to 88% acetonitrile over 26 min, at 0.25 mL min^−1^. After the LC phase, MS was carried out by electrospray ionization in the positive mode, with a capillary voltage of 3 kV and a cone voltage of 20 and 50 V. Detection and spectral characterization of the peaks was performed by photodiode array detection using Empower software v.3 (Waters) to extract bi-dimensional chromatograms at different wavelengths, normally within the range between 200 and 500 nm depending on the spectral characteristics of the desired compound.

### 2.4. Dereplication Studies (LC-DAD-HRMS)

The organic extracts of the co-culture were subjected to dereplication using a combination of LC-DAD-HRMS analysis with an Agilent 1200 Rapid Resolution HPLC system coupled to an ESI mode Bruker maXis mass spectrometer as described in [8]. The separation of compounds was conducted using acetonitrile and water containing ammonium formate 13 mM and 0.01% TFA as solvents in a Zorbax SB-C8 column (3.5 mm, 2.1 × 30 mm). The resulting chromatographic runs were processed using a Bruker in-house component extraction algorithm to identify Total Ion Chromatogram (TIC) positive peaks at 210 nm. For molecular formula interpretation, we used retention time and exact mass as search criteria on the high-resolution mass spectrometry database of the Medina Foundation (Granada, Spain). We look specifically for compounds with known antibiotic activity, such as undecylprodigiosin and manumycin A.

### 2.5. Growth Inhibition Bioassays

Cell extracts from the *Streptomyces* cultures were assessed for growth inhibition against *Staphylococcus aureus*. For the bioassays, 10 µL of cell extracts were deposited on sterile disks that had been previously placed on top of TSA (tryptone soya agar) plates inoculated with the corresponding indicator microorganism as follows. To grow the preinoculum of *S. aureus*, the cells were taken directly from a glycerol stock at −80 °C and inoculated in 20 mL of TSB (tryptone soya broth) medium and then grown until they reach an OD_600_ ~1. Then, 200 µL of the *S. aureus* cells were homogenized into 5 mL of tempered TSB (containing 0.7% of agar) and poured as a thin layer (5 mL) over a solid layer (20 mL) of TSB (containing 1.4% of agar) placed on the Petri dishes previously. After the solidification of the thin layer, the sterile disks were placed on the plates. In all cases, the same amount of ethyl acetate extracted sample (10 µL) was added to the disks. Antimicrobial activity was examined as visible inhibition halos after incubation of the agar plates for 24 h at 30 °C.

### 2.6. Keratinolytic Activity Detection Assay

Keratinolytic activity was determined following previous existing methods [13,14]. Liquid samples of 0.7 mL of each culture were taken every day and centrifuged for 20 min at 13,000 rpm to get a final 0.5 mL aliquot of supernatant. With the centrifugation step, cells and feather debris were removed. The 0.5 mL aliquot was mixed with 0.5 mL of 100 mM glycine-NaOH (pH 10) containing 1% casein and incubated at 37 °C for 20 min. In this reaction, tyrosine is released after the breaking of casein by the keratinases, among other enzymes. The reaction was stopped by the addition of 0.5 mL of trichloroacetic acid (20% *w*/*v*) and incubation for 15 min at room temperature. After the inactivation of the enzymes, the samples were centrifuged for 15 min at 13,000 rpm and the OD_280nm_ of the supernatant was measured with a spectrophotometer. A standard curve was performed using solutions of 0–700 mg L^−1^ of tyrosine. One keratinase unit (U) was defined as the amount of enzyme required to increase the absorbance by 0.01 unit (OD_280nm_) in one minute under the assay conditions employed.

## 3. Results

### 3.1. Characterization of Keratinolytic Activity in Streptomyces Albidoflavus AN1, Streptomyces Drozdowiczii AD1 and Streptomyces Griseoaurantiacus AD2

**Figure 1:**
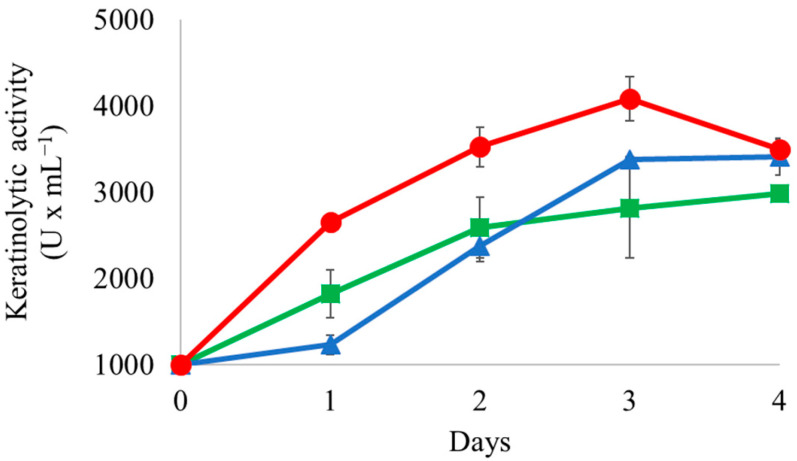
Keratinolytic activity of *Streptomyces albidoflavus* AN1 (green squares), *Streptomyces drozdowiczii* AD1 (blue triangles) and *Streptomyces griseoaurantiacus* AD2 (red circles) grown on CFMS (Chicken Feathers Mineral Salts) medium (pH = 8). Samples were collected every 24 h until cells reached the stationary phase to determine keratinolytic activity. Vertical error bars correspond to the standard error of the mean of three replicates.

In our recent work, we focused on the characterization of strains that can degrade chicken feathers [4,5]. Chicken feathers are considered waste and are normally dumped in landfills or incinerated [2]. Therefore, strains with the ability to hydrolyze such waste are very interesting from a biotechnological point of view. To follow up with this approach we decided to test the keratinolytic activity of the three *Streptomyces* species analyzed in this study. In order to characterize their keratinolytic activity, each strain was grown on CFMS (Chicken Feathers Mineral Salts) medium until reaching a stationary phase. Figure 1 shows the keratinolytic activity values that each of the strains displayed during the time course of the experiment. *S. griseoaurantiacus* AD2 showed the highest activity (~4000 U × mL^−1^); followed by the two other strains, which displayed a 25% lower activity (~3000 U × mL^−1^). In conclusion, the three strains have keratinolytic activity, which makes them promising candidates for biotechnological applications on feather valorization.

### 3.2. Effect of pH on the Keratinolytic Activity of the Strains

pH plays a major role in some cellular activities. In previous work, using similar assay conditions, we determined that the highest keratinolytic activity was achieved by *B. licheniformis* CG1 when the pH of the medium was 8 [5]. For this reason, the pH of the CFMS medium that we have developed for the study of the *Streptomyces* strains was set to 8 (see Materials and Methods section). However, to have a better insight into the conditions required by the strains to degrade keratin, we tested the effect of acid (pH = 6), neutral (pH = 7) and basic (pH = 8) pH on the keratinolytic activity of the three *Streptomyces* strains. The keratinolytic activity of each of the strains was determined in these conditions in the same way as in Section 3.1.

**Figure 2:**
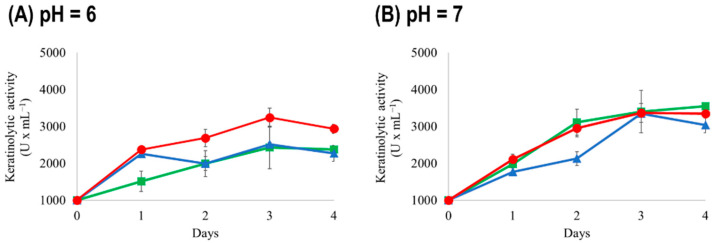
Keratinolytic activity of *Streptomyces albidoflavus* AN1 (green squares), *Streptomyces drozdowiczii* AD1 (blue triangles) and *Streptomyces griseoaurantiacus* AD2 (red circles) grown on CFMS (Chicken Feathers Mineral Salts) medium at two different pH. (**A**) pH = 6. (**B**) pH = 7. In all cases, samples were collected every 24 h and for 4 days to determine the keratinolytic activity. Vertical error bars correspond to the standard error of the mean of three replicated experiments.

The highest keratinolytic activity was detected at pH = 8 in all the strains, as for the *B. licheniformis* CG1 strain. Indeed, the keratinolytic activity decreased as follows: basic > neutral > acid (see Figure 1 and Figure 2). A similar trend was also noted for the keratinase activity of the *B. altitudinis* B12 strain, which was shown to be favored in basic conditions (i.e., pH from 8 to 10) [4]. We also checked the evolution of the pH due to chicken feather degradation and microbial metabolism from the start of the experiment (t = 0 h) to the end of the experiment (t = 96 h). As shown in Table 1, the final pH decreased to a value of ~6 in all the strains tested, independently of the initial pH (note that when the initial pH value was 6, the final pH was slightly decreased by ~0.2 units; see Table 1). In conclusion, acidification of the medium might decrease the keratinolytic activity of the strains, but only slightly since all showed a rather high keratinolytic activity in the range of pH tested.

### 3.3. Testing the Benefits of Co-Culturing the Strains in Terms of Antibiotic Production

**Figure 3:**
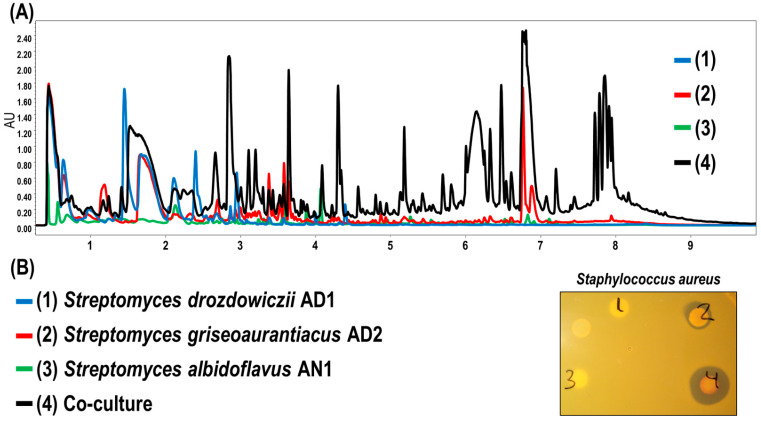
Analysis of metabolites and the antimicrobial activity of *Streptomyces albidoflavus* AN1, *Streptomyces drozdowiczii* AD1, *Streptomyces griseoaurantiacus* AD2 and the co-culture of the three species. (**A**) Comparative UPLC chromatograms of metabolites extracted with ethyl acetate from 96 h cultures of each of the strains and the co-culture of the three *Streptomyces* species in R5A medium. (**B**) Antimicrobial bioassay against *Staphylococcus aureus* with 10 μL of the cell extracts used for the UPLC analyses of section A that were extracted from the cultures shown in the figure.

We have previously shown that only *S. griseoaurantiacus* AD2 slightly inhibits the growth of some Gram-positive bacteria whereas the other two did not [8]. Therefore, we decided to assess the impact of co-culturing of *S. albidoflavus* AN1, *S. drozdowiczii* AD1 and *S. griseoaurantiacus* AD2 on the ability to produce antimicrobial activity in comparison with the antimicrobial activity of each strain grown individually. To do so, the strains were cultured (using the same amount of total starting inoculum) in R5A medium either separately or together for 4 days. Then, metabolites were extracted using ethyl acetate, and the resulting cell extracts were analyzed by UPLC analyses and antimicrobial bioassays (see Figure 3).

The UPLC analysis revealed that a significant number of peaks that were detected in the extracts of the co-culture were absent from the extracts of the single cultures (Figure 3A). Irrespective of which metabolites correspond to each of these peaks, the result clearly indicates that co-culturing of the strains highly increases metabolite production. Among these metabolites, undecylprodigiosin and manumycin A (with retention times around 7 min) have been previously shown to contribute to the antibiotic potential of *S. griseoaurantiacus* AD2 [8]. These two peaks are significantly increased in the cell extracts of the co-culture, in good correlation with the results of the antimicrobial bioassays against *S. aureus* shown in Figure 3B. The extracts from the co-culture and the culture of *S. griseoaurantiacus* AD2 exhibited antibiotic activity against the *Staphylococcus aureus* control strain, but the size of the inhibition halos was clearly bigger with the extracts of the co-culture, which indicates that the antibiotic activity is significantly increased when the three strains are cultured together (consistently with the results observed in the UPLC analyses). Therefore, these results highlight the benefits of co-cultivation of these bacterial species in terms of metabolic wealth and antibiotic potential.

### 3.4. Identification of Undecylprodigiosin and Manumycin A in the Cell Extracts of the Co-Culture

**Figure 4:**
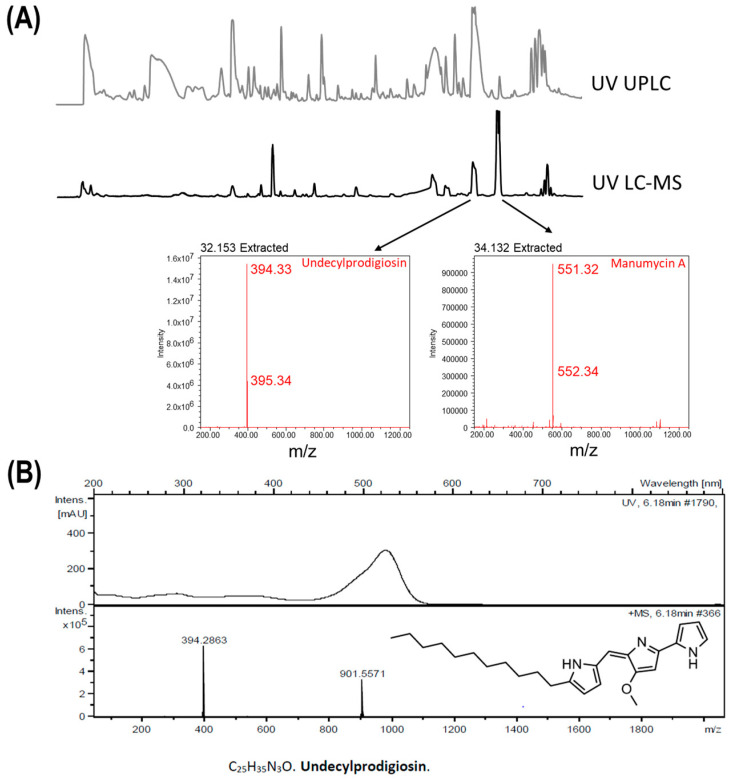
Identification of undecylprodigiosin and manumycin A metabolites present in the co-culture of *Streptomyces albidoflavus* AN1, *Streptomyces drozdowiczii* AD1 and *Streptomyces griseoaurantiacus* AD2 by LC-MS and LC-DAD-HRMS dereplication analyses. (**A**) UV chromatograms of both UPLC (grey) and LC-MS (black) analyses and MS analyses of the extracted UV peaks indicated by arrows; resulting in the identification of the metabolites manumycin A and undecylprodigiosin. (**B**) Identification of undecylprodigiosin by LC-DAD-HRMS dereplication analyses.

To confirm the synthesis of undecylprodigiosin and manumycin A in the co-culture condition both LC-MS and LC-DAD-HRMS analyses were performed. In the late retention times of the UV chromatograms, the LC-MS analysis allowed the identification of two metabolites with masses of *m*/*z* 394.33 [M + H]^+^ and *m*/*z* 551.32 [M + H]^+^, which correspond to the masses of undecylprodigiosin (C_25_H_35_N_3_O) and manumycin A (C_31_H_38_N_2_O_7_), respectively (Figure 4A). By LC-DAD-HRMS dereplication analyses these two metabolites were further confirmed to be undecylprodigiosin (Figure 4B) and manumycin A or D (depending on the number of Hydrogens in the formula; i.e., H_38_ or H_40_, respectively.

### 3.5. Using Chicken Feathers as Sole Substrate for Growing the Streptomyces Strains

Finally, we checked the capability of the individual strains and the co-culture (since it provides an advantage in terms of metabolic wealth) to use chicken feathers as the sole nutrient source for their growth. To do so, we developed a medium that we named CFTW (Chicken Feathers Tap Water medium). This medium contains only tap water and chicken feathers, which makes its production cheap and thus well adapted for the development of a microbial recycling platform for the valorization of keratin-rich waste. In preliminary analyses, we checked that each of the strains was able to grow on chicken feathers as their sole nutrient source (either using tap water or distilled water), but none of the cultures (including the co-culture) produced the characterized reddish color of the *S. griseoaurantiacus* AD2 cultures in R5A (Supplementary Figure S1. This result indicates that both tap water and distilled water can be employed for growing the strains (however, for a biotechnological application tap water would provide an economic advantage). In agreement with the obtained phenotypes (no red antibiotic is produced), we observed that the antibiotic activity of both the co-culture and the *S. griseoaurantiacus* AD2 culture was decreased in comparison with the R5A cultures (see Figure 3B and Figure S1). Supplementary Materials Figure S1 also shows that the speed of growth of the co-culture was significantly higher than that of the most efficient keratin-degrading strain (i.e., *S. griseoaurantiacus* AD2), which indicates that, not only antimicrobial activity is increased in the co-culture, but also the efficiency in the chicken feathers degradation.

**Figure 5:**
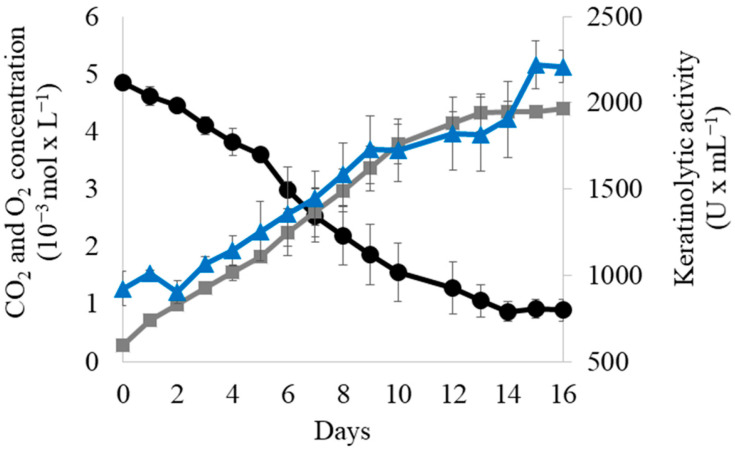
Growth and keratinolytic activity of *Streptomyces albidoflavus* AN1, *Streptomyces drozdowiczii* AD1 and *Streptomyces griseoaurantiacus* AD2 co-culture on chicken feathers as the sole substrate. Samples for growth (CO_2_ production and O_2_ consumption) and keratinolytic activity were collected every 24 h until O_2_ was depleted from the bottles. Blue triangles (keratinolytic activity), Grey squares (CO_2_ production) and Black circles (O_2_ consumption). Vertical error bars correspond to the standard error of the mean of two replicated experiments.

As a final stage, we decided to measure the keratinolytic activity of the co-culture during a time-course experiment. Figure 5 shows the growth and keratinolytic activity of the co-culture during the time-course experiment. Although the strains grew slower and produced lower keratinolytic activity values than when cultured in CFSM medium (compare Figure 5 with Figure 1 and Figure 2), their ability to grow on chicken feathers as their sole nutrient source is very promising for future microbial recycling strategies.

## 4. Discussion

In this study, we characterized three *Streptomyces* species with high keratinolytic activity. One of these strains (*S. griseoaurantiacus* AD2) showed a maximal keratinolytic activity of 4000 U × mL^−1^, whereas the activity displayed by the other two (*S. albidoflavus* AN1 and *S. drozdowiczii* AD1) was a little lower (~3000 U × mL^−1^). Very few strains with keratinolytic activity higher than 4000 U × mL^−1^ were reported in the literature [5], therefore *S. griseoaurantiacus* AD2 could be considered one of the most efficient keratin-degrading microorganisms discovered to date. At the same time, *Streptomyces* species are the most important antibiotic producers discovered to date [15,16,17]. *S. griseoaurantiacus* AD2 is also able to inhibit the growth of Gram-positive bacteria, which identifies this species as a very promising candidate for the valorization of keratin-rich wastes.

Most importantly, we have shown that a co-culture of the three *Streptomyces* strains led to greater keratinolytic and antibiotic activities than mono-cultures of each individual strain. All the strains were able to grow individually on chicken feathers as the sole nutrient and energy source, but the co-culture of these strains produced higher antimicrobial activity and faster growth on this substrate. Many reports in the literature describe the interesting effects of inter- or intra-species co-cultivation on different metabolic properties but the causes behind these impacts remain unfortunately unknown. Nevertheless, this approach could prove useful to valorize keratin-rich wastes (such as chicken feathers) as a cheap way to produce antibiotics. The ability to grow on a medium made just with water and chicken feathers (with no other types of requirements) would make the growth of these bacterial species very cheap for industrial production platforms. Another possible utilization of these species is directly as biofertilizers and/or biopesticides in sustainable agriculture. Indeed, most *Streptomyces* species are soil-borne bacteria and live in symbiosis with plants and other microorganisms [6]. They are known to have a plant growth-promoting effect [18] and to contribute to disease-suppressive soils [6]. Although much is still to be discovered concerning the colonization strategies and molecular interactions between plant roots and these bacteria, they are destined to become important players in modern sustainable agriculture [19,20].

In conclusion, this work constitutes a first step in the development of microbial strategies useful for the development of various biotechnological applications related to the utilization of keratin wastes as biofertilizers or as a cheap substrate to produce antibiotics.

## Figures and Tables

**Table 1:** pH evolution from the initial value to the final value reached after 4 days of growth of the strains *S. drozdowiczii* AD1, *S. griseoaurantiacus* AD2 and *S. albidoflavus* AN1.

	Initial pH t = 0 h	Final pH t = 96 h	Initial pH t = 0 h	Final pH t = 96 h	Initial pH t = 0 h	Final pH t = 96 h
AD1	8.0	6.2 ± 0.2	7.0	6.2 ± 0.1	6.0	5.9 ± 0.1
AD2	8.0	6.0 ± 0.1	7.0	6.0 ± 0.1	6.0	5.8 ± 0.1
AN1	8.0	6.0 ± 0.1	7.0	6.0 ± 0.1	6.0	5.7 ± 0.2

## Data Availability

The authors declare that all data obtained have been included in the manuscript, its additional files and/or repositories.

## Supplementary Materials

The following supporting information can be downloaded at: https://www.mdpi.com/article/10.3390/microorganisms11051109/s1, Figure S1: Comparison of growth and antimicrobial activity between *Streptomyces griseoaurantiacus* AD2 and the co-culture on chicken feathers as sole nutrient source.
