# Supplementary material for: Characterization of the Keratinolytic Activity of Three Streptomyces Strains and Impact of Their Co-Cultivation on This Activity
Source: Microorganisms. 2023 Apr 24;11(5):1109. doi: 10.3390/microorganisms11051109 (PMC10222057; doi:10.3390/microorganisms11051109)
Supplement: Supplementary file 1 [file microorganisms-11-01109-s001.zip › microorganisms-2329957-supplementary.pdf]

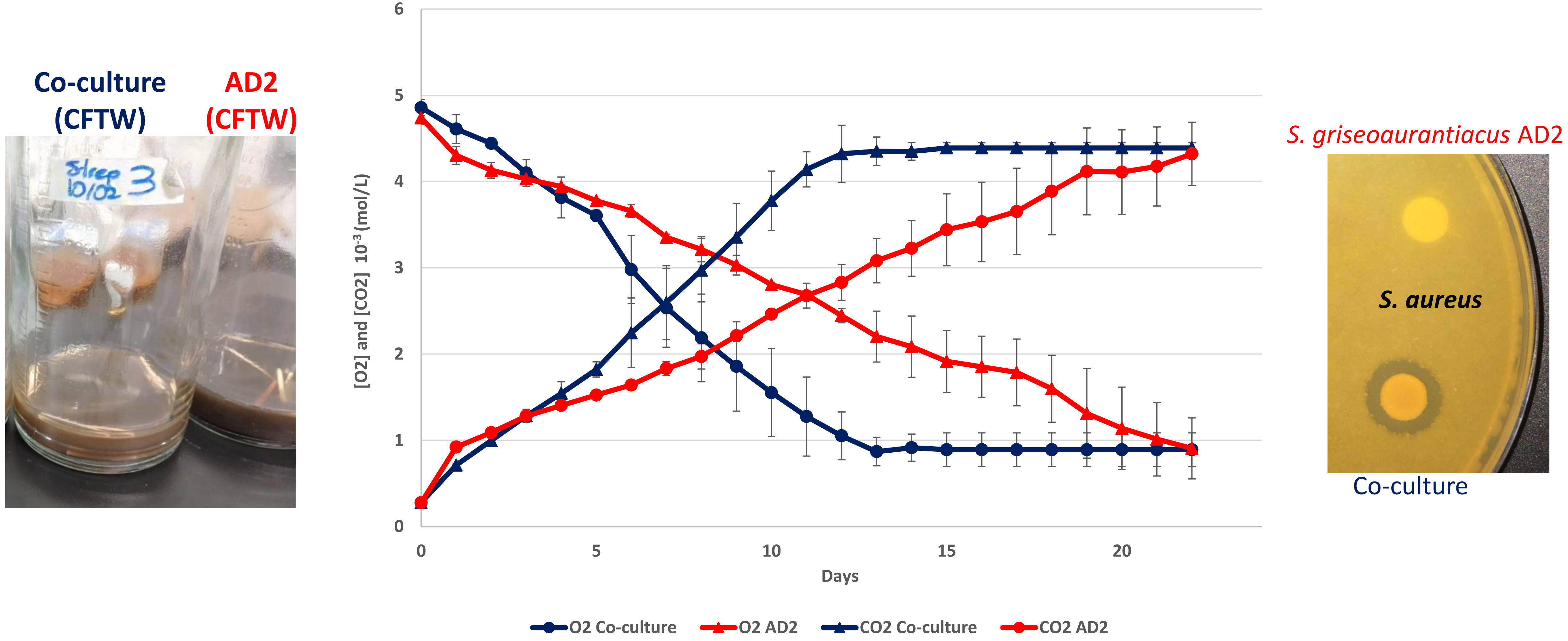

Supplementary Figure S1. Comparison of growth and antimicrobial activity between *Streptomyces griseoaurantiacus* AD2 and the co-culture on chicken feathers as sole nutrient source. Samples for growth (CO_2 production and O_2 consumption) were collected every 24 hours until O_2 was depleted from the AD2 bottles. Vertical error bars correspond to the standard error of the mean of two replicated experiments. The phenotype of the cultures on CFTW medium and the antimicrobial bioassays against *Staphylococcus aureus* with the cells extracts of the cultures at the end of the time series experiment are also shown in the figure.
